# 6-Gingerol Alleviates Ferroptosis and Inflammation of Diabetic Cardiomyopathy via the Nrf2/HO-1 Pathway

**DOI:** 10.1155/2022/3027514

**Published:** 2022-12-31

**Authors:** Shenglin Wu, Jinxiu Zhu, Guihai Wu, Zuoqi Hu, Pengxiang Ying, Zhijun Bao, Zipeng Ding, Xuerui Tan

**Affiliations:** ^1^Institute of Clinical Electrocardiology, The First Affiliated Hospital of Shantou University Medical College, Shantou, 515041 Guangdong, China; ^2^Longgang Maternity and Child Institute of Shantou University Medica College, Shenzhen 518100, Guangdong, China; ^3^Clinical Research Center, The First Affiliated Hospital of Shantou University Medical College, Shantou 515041, Guangdong, China

## Abstract

**Background:**

Diabetes mellitus (DM) can induce cardiomyocyte injury and lead to diabetic cardiomyopathy (DCM) which presently has no specific treatments and consequently increase risk of mortality.

**Objective:**

To characterize the therapeutic effect of 6-gingerol (6-G) on DCM and identify its potential mechanism.

**Methods:**

In vivo streptozotocin- (STZ-) induced DM model was established by using a high-fat diet and STZ, followed by low-dose (25 mg/kg) and high-dose (75 mg/kg) 6-G intervention. For an in vitro DCM model, H9c2 rat cardiomyoblast cells were stimulated with high glucose (glucose = 33 mM) and palmitic acid (100 *μ*M) and then treated with 6-G (100 *μ*M). Histological and echocardiographic analyses were used to assess the effect of 6-G on cardiac structure and function in DCM. Western blotting, ELISA, and real-time qPCR were used to assess the expression of ferroptosis, inflammation, and the Nrf2/HO-1 pathway-related proteins and RNAs. Protein expression of collagen I and collagen III was assessed by immunohistochemistry, and kits were used to assay SOD, MDA, and iron levels.

**Results:**

The results showed that 6-G decreased cardiac injury in both mouse and cell models of DCM. The cardiomyocyte hypertrophy and interstitial fibrosis were attenuated by 6-G treatment in vivo and resulted in an improved heart function. 6-G inhibited the expression of ferroptosis-related protein FACL4 and the content of iron and enhanced the expression of anti-ferroptosis-related protein GPX4. In addition, 6-G also diminished the secretion of inflammatory cytokines, including IL-1*β*, IL-6, and TNF-*α*. 6-G treatment activated the Nrf2/HO-1 pathway, enhanced antioxidative stress capacity proved by increased activity of SOD, and decreased MDA production. Compared with in vivo, 6-G treatment of H9c2 cells treated with high glucose and palmitic acid could produce a similar effect.

**Conclusion:**

These findings suggest that 6-G could protect against DCM by the mechanism of ferroptosis inhibition and inflammation reduction via enhancing the Nrf2/HO-1 pathway.

## 1. Introduction

Diabetes mellitus (DM) affects more than 8.5% of the population worldwide [1]. Diabetic cardiomyopathy (DCM) increases the mortality of the diabetics by contributing to heart failure [2]. DCM, a distinct myocardial disease induced by DM, is characterized by both structural and functional changes, including apoptosis, interstitial fibrosis, and diastolic dysfunction [3, 4]. Despite the increase in basic and clinical studies on DCM over the past few decades, its mechanisms still remain unclear.

The serum levels of the advanced glycation end products (AGEs) are increased along with the increased level of blood glucose [5], and energy metabolism in cardiomyocytes shifts from glycogen decomposition to fatty acid oxidation, resulting in intracellular lipid accumulation and increased lipid toxicity [6]. The accumulation of AGEs and lipids causes excessive production of reactive oxygen species (ROS), which ultimately leads to DCM [7]. The sustained accumulation of ROS increases the expression of several proinflammatory factors, including IL-1*β*, IL-6, and TNF-*α* [8], and elevated level of IL-6 can predict the development of type 2 DM [9].

Ferroptosis is a new form of cell necrosis mediated by iron-dependent lipid peroxidation [10]. Ferroptosis has been related to the mechanism associated with lipopolysaccharide-induced cardiac dysfunction and cardiac ischemia/reperfusion injury [11, 12]. Although these changes have been observed in acute injury disease models of the heart, it is not clear whether ferroptosis also involved in myocardial damage in DCM. Studies show that iron overload in patients with DM not only increases the risk of DM progression and insulin resistance but also exacerbates cardiovascular complications through the Fenton reaction [13, 14]. Therefore, it is important to clarify whether ferroptosis plays a role in the pathogenesis of DCM. Moreover, inflammation is also an important pathological mechanism for the occurrence and progress of DCM [15]. Based on the above, antiferroptosis and anti-inflammatory therapy are considered potential strategies for DCM.

Nowadays, the use of traditional Chinese medicine to treat DCM has attracted people's attention. Ginger was used to treat diseases in ancient China. 6-Gingerol (6-G), a polyphenol extracted from ginger, has been reported to have anti-inflammatory, antiapoptotic, and antioxidant properties [16–18], and studies have shown that 6-G can upregulate the nuclear factor erythroid 2-related factor 2 (Nrf2) pathway to alleviate liver injury [19, 20]. 6-G can also modulate ferroptosis by inhibiting ubiquitin-specific peptidase 14 [21]. Moreover, 6-G is reported to alleviate DCM [22]. However, the exact mechanism of 6-G treatment in DCM remains unclear. Therefore, we propose that 6-G protects against DCM by alleviating ferroptosis and inflammation through Nrf2/HO-1. To test this, we observed the effects of 6-G on DCM in mouse and H9c2 cell DCM models and explored its underlying mechanism.

## 2. Materials and Methods

### 2.1. Chemicals

6-G was purchased from the Chengdu Herbpurify Co., Ltd. (Chengdu, China) with a purity > 98% tested by high-performance liquid chromatography.

### 2.2. Animals and Treatments

Four- to five-week-old male C57BL/6 mice were purchased from Charles River (Beijing, China). The DCM model was implemented by placing mice on a high-fat (D12492, Research Diet) diet for 4 weeks, followed by overnight fasting and intraperitoneal injection with a dose of streptozotocin (STZ) (50 mg/kg × 5 days) dissolved in pH 4.5 sodium citrate buffer. After one week, mice with blood glucose higher than 16.7 mmol/L were considered as having DM [23] and continuous high-fat feeding. Control mice were given a normal diet and treated with sodium citrate buffer and continuous normal diet feeding. Mice were divided into five groups as follows: control (CON): control mice were treated with corn oil; 6-G-treated (6-G): control mice were treated with 6-G (75 mg/kg) dissolved in corn oil; STZ-treated (STZ): DM mice were treated with corn oil; STZ+25 mg/kg 6-G-treated (STZ+6-G L): DM mice were treated with 6-G (25 mg/kg) dissolved in corn oil; and STZ+75 mg/kg 6-G-treated (STZ+6-G H): DM mice were treated with 6-G (75 mg/kg) dissolved in corn oil. The corn oil and 6-G with corn oil were administered by intragastric feeding, and all groups were treated for 12 weeks. All mice were implemented euthanasia via CO_2_. All experimental procedures were approved by The Institutional Animal Research and Use Committee of Shantou University (animal protocol approval number: SUMC2020-386).

### 2.3. Echocardiography

Echocardiography was used to evaluate mouse cardiac function by ultrasound professionals with a multimode imaging system (Fujifilm, Canada). All mice were anesthetized with 0.5% isoflurane, followed by chest hair removal. We obtained images of the parasternal short axis under M-mode and averaged the left ventricle measurements from five continuous cardiac cycles, including the left ventricle end-diastolic dimension (LVEDd), left ventricle end-systolic dimension (LVEDs), left ventricular diastolic diameter (LVIDd), left ventricular systolic diameter (LVIDs), end-diastolic left ventricle posterior wall thickness (LVPWd), and end-systolic left ventricle posterior wall thickness (LVPWs). Finally, the left ventricle ejection fraction (EF) and fractional shortening (FS) were calculated based on the above indicators.

### 2.4. Histology

The hearts were harvested after 6-G treatment for 12 weeks, and the control group was sampled at the same time. The excess blood was removed by perfusion with 10% KCl. The tissue was cut into 4-5 *μ*m sections after being fixed with 4% paraformaldehyde and embedded in paraffin. Hematoxylin and eosin (HE) staining was used to assess the cardiomyocyte cross-sectional area and to trace the outline of striated muscle. A Prussian Blue Stain kit (Solarbio, G1424, China) was used to assess the level of iron. Collagen I and collagen III were detected by immunohistochemistry to evaluate interstitial fibrosis. Briefly, antigen unmasking was performed with citrate buffer (0.01 M, pH = 6.0) in the microwave; then, a ready-to-use immunohistochemical hypersensitivity Ultrasensitive™ SP kit (KIT-9710, MXB, China) was used to block nonspecific binding sites, followed by overnight incubation with antibodies against collagen I (1 : 100, Abcam, ab7778) and collagen III (1 : 100, Abcam, ab34710) at 4°C. Sections were washed with PBS 3 times, 10 min each wash, and then incubated with anti-rabbit secondary antibodies from the kit. Finally, staining was visualized with DAB followed by counterstaining with hematoxylin after washing with PBS. Image-Pro Plus 6.0 was used to analyze the photos.

### 2.5. Cell Culture

H9c2 cells were purchased from the Cell Bank of the Chinese Academy of Sciences (Shanghai, China) and were cultured in high-glucose DMEM (C1195, Gibco, USA) with 10% fetal bovine serum (10270, Gibco), streptomycin (100 mg/mL), and penicillin (100 U/mL) (15140, Gibco). DCM cell model (high-glucose group) was constructed by 33 mM glucose and 100 mM palmitic acid. Cells were divided into four groups: control (CON), 6-G treated (6-G, 100 *μ*M), high glucose (HG), and HG+6-G (HG+6-G, 100 *μ*M).

### 2.6. Quantitative Real-Time PCR (RT-PCR)

Cardiac tissue was immersed in TRIzol (TIANGEN, China) and ground in a grinding machine (JXFSTPRP-CL, Jingxin, China) to extract total RNA. RNA reverse transcription was performed with a PrimeScript™ RT with a gDNA Eraser kit (RR047A, TAKARA). Amplification reactions were performed on a real-time PCR thermocycler (Bio-Rad, USA) with TB Green® Premix Ex Taq™ II (RR820A, TAKARA). GAPDH gene expression was used for normalization. All primers used in this study are listed in Table 1.

### 2.7. Western Blotting

Cardiac tissues were immediately placed in liquid nitrogen after sampling and then stored in a refrigerator at -80°C. All heart samples were immersed in lysis buffer and then grounded in a grinder. The Pierce BCA Protein Assay kit (23225, Thermo, USA) was used to measure protein concentration. Protein (40 *μ*g) was separated by gel electrophoresis and transferred to polyvinylidene difluoride (PVDF) membranes; then, membranes were blocked with 5% milk in Tris-buffered Tween 20 for 2 h and then incubated overnight with primary antibody at 4°C. The following day, PVDF membranes were immersed in secondary antibody (1 : 10000) (ab6721, Abcam) for 2 h. Finally, protein bands were visualized by ECL working solution, and band intensity was quantified with Image Lab. In this study, primary antibodies were as follows: HO-1 (1 : 2000) (ab13243, Abcam), Nrf2 (1 : 1000) (16396-1-AP), GPX4 (1 : 5000) (ab125066, Abcam), FACL4 (1 : 1000) (ab155282, Abcam), and GAPDH (1 : 10000) (ab181602, Abcam).

### 2.8. IL-6, IL-1*β*, and TNF-*α* ELISA

IL-6, IL-1*β*, and TNF-*α* levels in serum were detected by ELISA kits (H007, H002, and H052, Nanjing Jiancheng, China), according to the manufacturer's instructions.

### 2.9. Superoxide Dismutase (SOD) and Malondialdehyde (MDA)

Myocardial tissues were ground as the following protocol: 9 mL 0.9% physiological saline per 1 g tissue. Tissue homogenate supernatant was collected after centrifugation at 12,000 rpm for 15 min. H9c2 cells were scraped off the Petri dish with a cell scraper, and suspended in a reagent was added to make the suspension; then, ultrasonic lysis was performed. The levels of SOD (A001-3-2, Nanjing Jiancheng, China) and MDA (A003-1-2, Nanjing Jiancheng, China) were detected by using corresponding commercial test kits.

### 2.10. CCK-8 Assay

A Cell Counting Kit-8 (CCK-8) (HANBIO, China) was used to assess cell viability. Cells were placed in a 96-well plate (5000 cells per well) and incubated for 12 h in 5% CO_2_ at 37°C. Then, different concentrations of 6-G were added to the plate, and cells were further cultured for 24 h. Then, 10 *μ*L CCK-8 was added to each well, and absorbance at 450 nm was monitored by a microplate reader (BioTek, USA).

### 2.11. Statistical Analysis

All values are expressed as mean ± SEM, and a one-way ANOVA test followed by a post hoc Tukey test was used to determine statistical significance. All analyses were performed in SPSS 19.0 statistical software. A *p* < 0.05 was considered to indicate statistical significance.

## 3. Results

6-G improves cardiac function and reduces pathological changes of DCM.

Blood glucose in the STZ group was higher than in the CON group and decreased slightly after 6-G (75 mg/kg) treatment (Table 2). Compared with the CON group, heart weight (HW), lung weight (LW), HW/body weight (BW), and LW/BW in the STZ group increased compared with the CON group. The 6-G treatment prevented the increase in HW, LW, HW/BW, and LW/BW of diabetic mice. The BW of the STZ group did not decrease, which may be related to the high-fat diet.

**Figure 1:**
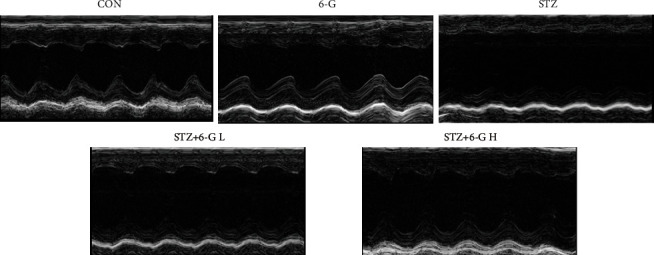
Representative echocardiographic pictures of each group.

As shown in Table 3 and Figure 1, the cardiac function of the STZ group was decreased compared with the CON or CON +6-G groups, including LVPWd, LVPWs, LVIDs, LVIDs, EF, and FS, and all of these parameters could be restored by 6-G treatment.

### 3.1. M-Mode Imaging of the LV Midpapillary Level in the Parasternal Short-Axis View

**Figure 2:**
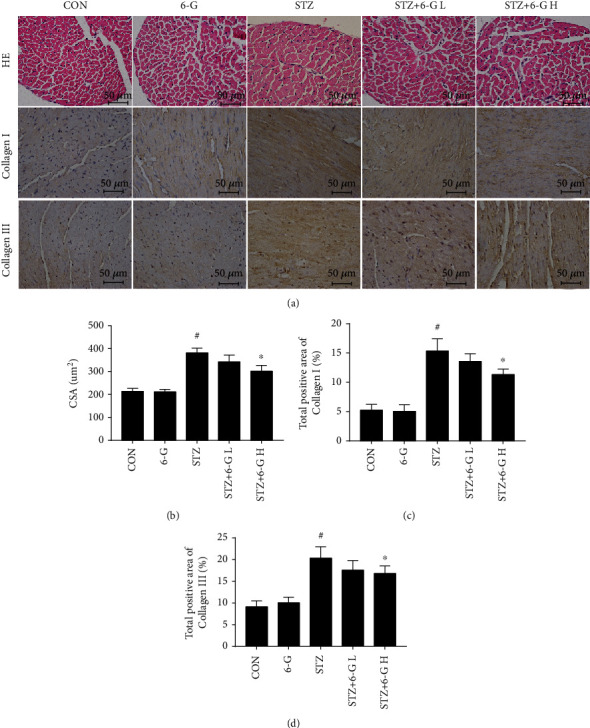
6-Gingerol prevents STZ-induced mouse heart remodeling and fibrosis. (a) Hematoxylin and eosin (HE) staining showing the morphological changes of myocardial tissue (*n* = 6). Immunohistochemistry shows the expression of collagen I/III (*n* = 6). (b) Cross-sectional area (CSA) of cardiomyocytes (*n* = 6). (c, d) The positive area of collagens I and III (*n* = 6). All values are expressed as mean ± SEM. ^#^*p* < 0.05, compared with the CON or 6-G group; ^∗^*p* < 0.05, compared with the STZ group.

HE staining showed that, compared with the CON group, STZ treatment increased the cross-sectional area of the cardiomyocytes, but this increase could be attenuated by 6-G treatment (Figures 2(a) and 2(b)). Immunohistochemical staining for collagens I and III indicated interstitial fibrosis increased in the STZ group but could be reduced by 6-G treatment (Figures 2(a), 2(c), and 2(d)).

### 3.2. 6-G Blocks Cardiomyocyte Ferroptosis and Inflammation in DCM

**Figure 3:**
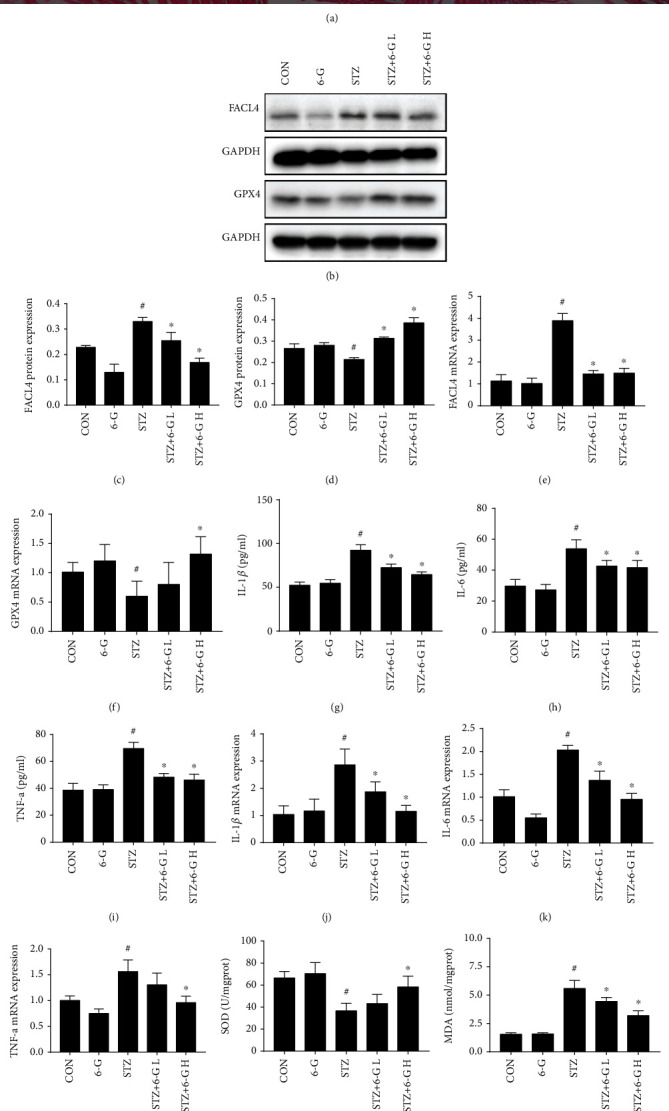
6-Gingerol attenuates STZ-induced ferroptosis and inflammation in vivo. (a) Prussian blue staining showing the level of iron ion. (b) Representative blots of FACL4 and GPX4 in the mouse heart (*n* = 6). (c, d) Histograms showing the fold change of FACL4 and GPX4. (e, f) The relative mRNA expression of FACL4 and GPX4 (*n* = 4); IL-1*β* (g), IL-6 (h), and TNF-*α* (i) activities in mouse serum (*n* = 6). Relative mRNA expression of IL-1*β* (j), IL-6 (k), and TNF-*α* (l) (*n* = 4). Heart tissue levels of SOD (m) and MDA (n) in mice. All data for proteins and mRNA was normalized to GAPDH before relative quantitative analysis. All values are expressed as mean ± SEM. ^#^*p* < 0.05, compared with the CON or 6-G group; ^∗^*p* < 0.05, compared with the STZ group.

The content of iron in the STZ group was higher than those in the CON and 6-G groups, and 6-G treatment decreased its level (Figure 3(a)). Moreover, the lipid peroxidation index also changed correspondingly in vivo. SOD enzymatic activity was decreased in DCM and could be reversed by 6-G treatment (Figure 3(m)), whereas the level of MDA presented the opposite change (Figure 3(n)). Compared with the CON and 6-G groups, protein and mRNA levels for proferroptotic FACL4 were upregulated in the STZ group but could be downregulated by 6-G treatment (Figures 3(b), 3(c), and 3(e)). Protein and mRNA levels for antiferroptotic glutathione peroxidase-4 (GPX4) showed the opposite trend (Figures 3(b), 3(d), and 3(f)). Inflammatory markers also showed changes similar to FACL4.

The serum protein level of IL-1*β*, IL-6, and TNF-*α* was lower in control mice compared to the diabetic mice and could be reduced in diabetic mice after 6-G treatment (Figures 3(g)–3(i)). Similar to the results in serum, the mRNA levels of IL-1*β*, IL-6, and TNF-*α* in heart tissue were upregulated in diabetic mice but could be reduced by 6-G treatment (Figures 3(j)–3(l)).

### 3.3. 6-G Inhibits the Ferroptosis and Inflammation in H9c2 Cells

**Figure 4:**
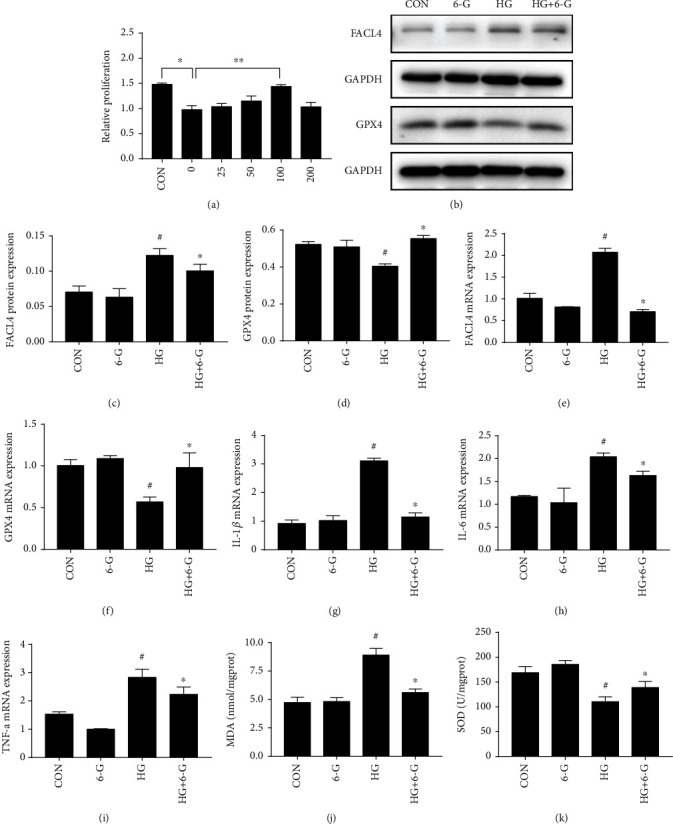
6-Gingerol inhibits ferroptosis and inflammation in vitro. (a) Cell proliferation of H9c2 in different concentrations of 6-G was determined by a CCK-8 cell proliferation assay. (b) Representative blots of FACL4 and GPX4 in H9c2 cells (*n* = 4). (c, d) Histograms showing the fold change of FACL4 and GPX4. Relative mRNA expression of FACL4 (e), GPX4 (f), IL-1*β* (g), IL-6 (h), and TNF-*α* (i) (*n* = 4). All data for protein and mRNA levels were normalized to GAPDH before quantitative analysis. All values are expressed as mean ± SEM. ^#^*p* < 0.05, compared with the CON or 6-G group; ^∗^*p* < 0.05, compared with the HG group.

We first established a palmitic acid and HG-induced H9c2 model to simulate DCM according to the results of the previous experiments. To explore the effect of different concentrations of 6-G on ferroptosis and inflammation of palmitic acid and HG induced the cell model of DCM, we treated palmitic acid and HG-induced H9c2 cells with different concentrations of 6-G (0, 25, 50, 100, and 200 *μ*M). Cell proliferation was evaluated by CCK-8 to obtain the optimal 6-G concentration (Figure 4(a)). Results suggested that a suitable concentration of 6-G exhibits a protective effect on palmitic acid and HG-stressed H9c2 cells.

Compared with the CON and 6-G groups, FACL4 protein and mRNA levels were upregulated in the HG group induced by palmitic acid and HG and downregulated by 6-G treatment (Figures 4(b), 4(c), and 4(e)). GPX4 protein and mRNA levels showed the opposite trend (Figures 4(b), 4(d), and 4(f)). Consistent with lipid peroxides in vivo, HG-mediated increase in MDA could be decreased by 6-G treatment, while SOD showed the opposite trend (Figures 4(j) and 4(k)). At the same time, the mRNA levels of IL-1*β* (Figure 4(g)), IL-6 (Figure 4(h)), and TNF-*α* (Figure 4(i)) were increased in the HG group, compared with the CON group, and decreased after 6-G treatment (Figures 4(g)–4(i)).

### 3.4. 6-G Enhances the Nrf2/HO-1 Pathway in DCM Both In Vivo and In Vitro

**Figure 5:**
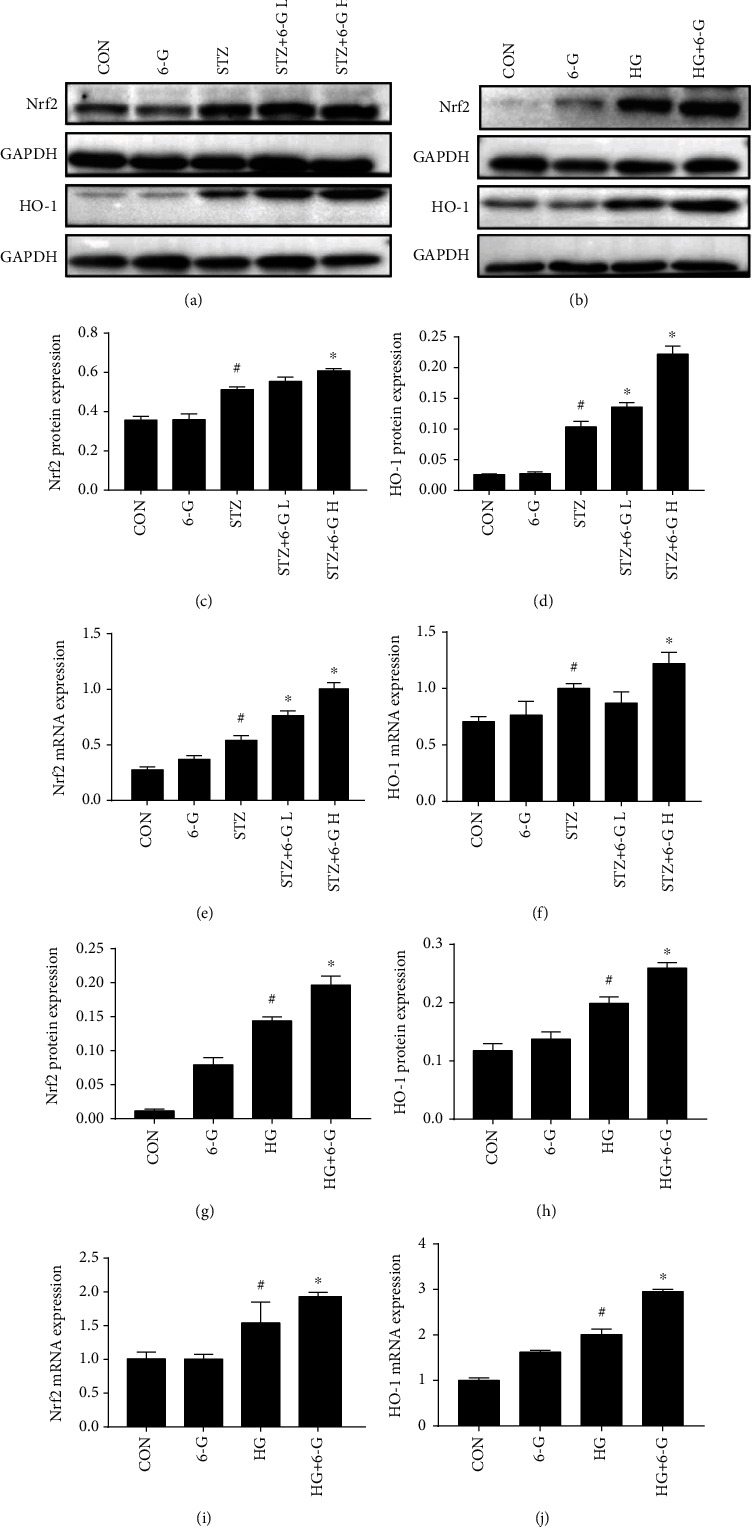
6-G upregulates the expression of Nrf2 and HO-1 in DCM both in vivo and in vitro. (a) Representative blots for Nrf2 and HO-1 in the mouse heart (*n* = 6). (b) Representative blots of Nrf2 and HO-1 in H9c2 cells (*n* = 4). (c, d) Histograms showing the fold change of Nrf2 and HO-1 in mouse hearts. (e, f) Relative mRNA expression of Nrf2 and HO-1 in mouse hearts (*n* = 6). (g, h) Histograms showing the fold change of Nrf2 and HO-1 in H9c2 cells. (i, j) Relative mRNA expression of Nrf2 and HO-1 in H9c2 cells (*n* = 4). All data for proteins and mRNA was normalized to GAPDH before relative quantitative analysis. All values are expressed as mean ± SEM. ^#^*p* < 0.05, compared with the CON or 6-G group; ^∗^*p* < 0.05, compared with the HG or STZ group.

In vivo experiments, STZ elevated the expression of Nrf2 and HO-1 at both the mRNA and protein levels, and 6-G treatment caused a further increase (Figures 5(a) and 5(c)–5(f)). High glucose upregulated the expression of Nrf2 and HO-1 in H9c2 cells and significantly upregulated after 6-G treatment (Figures 5(b) and 5(g)–5(j)).

## 4. Discussion

It has been shown that inflammation and oxidative stress increase in heart tissue of STZ-induced DM mice, which leads to cardiac fibrosis, cardiomyocyte ferroptosis, apoptosis, and finally cardiac dysfunction [8, 24]. 6-G is a natural phenolic that could exert anti-inflammatory, antitumor, and antioxidant effects [18, 21, 25]. However, how 6-G decreases the risk of DCM remains unknown. We used a mouse model and cell model to uncover the underlying mechanisms and found that 6-G treatment effectively promotes Nrf2 expression and enhances the translocation of genes encoding antioxidant enzymes accordingly, such as HO-1 and SOD. In addition, our study shows that 6-G treatment inhibits ferroptosis, inflammation, and cardiac fibrosis to reduce the damage that occurs in DCM.

### 4.1. 6-G Attenuates Ferroptosis in DCM

Our study showed that ferroptosis increases in DCM mice and HG and palmitic acid treated H9c2 cells. This is consistent with the results of previous studies [24, 26]. Studies have shown that a connection between metal toxicity and diabetic complications, specifically, reduced iron content could attenuate DCM injury [27, 28]. DCM is characterized by overproduction of ROS [29]. As mentioned earlier, iron homeostasis imbalance and lipid peroxidation are two major factors of ferroptosis [10]. When iron homeostasis is disrupted, excessive free iron leads to an increase in the production of ROS by the Fenton reaction, thus inducing lipid peroxidation-mediated cell death [30]. In this study, we found that FACL4 is increased in DCM mice, and 6-G treatment can prevent the increase. FACL4 controls the activity of the iron-containing enzyme lipoxygenase, which is a central promoter of ferroptosis due to its ability to produce lipid hydroperoxides [31]. Not only that, but it also dictates sensitivity versus resistance to ferroptosis [32]. GPX4 is decreased in DCM mice, but it can be partially restored through 6-G treatment. GPX4 attenuates lipid peroxide toxicity and maintains lipid bilayer homeostasis [33]. Under normal circumstances, the body has enough GPX4 to withstand oxidative stress. The body's antioxidant system is damaged, and GPX4 is decreased when DCM occurs. The increase in the level of GPX4, by 6-G, to reduce oxidative stress is probably why 6-G reduces ferroptosis in DCM.

### 4.2. 6-G Attenuates Inflammatory in DCM

Inflammation is increased in DCM in vitro and vivo, and 6-G treatment reduces this inflammation. This confirms previous studies showing that inflammatory cytokines IL-1*β*, IL-6, and TNF-*α* are elevated in DCM [34, 35]. 6-G also has anti-inflammatory effects in other diseases such as sepsis, cerebral ischemia/reperfusion injury, and diabetic nephropathy [25, 36, 37]. The protein levels and mRNA levels of inflammatory factors IL-1*β*, IL-6, and TNF-*α* are increased in DCM in vitro and in vivo, and 6-G treatment can reduce this increase.

### 4.3. The Nrf2/HO-1 Pathway Is Active by 6-G in DCM

6-G may regulate ferroptosis and inflammation through Nrf2/HO-1 pathway. A conflict is observed in the expression of Nrf2 in DCM, some are increased [38], and some are decreased [8], which may account for the different models used. A study showed that in DM, the Nrf2 pathway is activated in obese mice and inactivated in nonobese mice [39]. We show DCM mice given a high-fat diet, to maintain the status of diabetes, have increased Nrf2. This is also the reason why the BW of mice in the STZ group increases rather than decreases. A prior study obtained similar results showing Nrf2 is upregulated by a high-fat diet [40]. In addition, activation of the Nrf2 pathway can reduce inflammation. The expression of inflammation-associated cytokines IL-1*α*, IL-12*β*, CSF2, IL-6, and TNF-*α* is increased in Nrf2^−/−^ mice after LPS injection in Nrf2^−/−^ [41]. Sulforaphane prevents ferroptosis in DCM via the AMPK/Nrf2 pathway [26]. We propose that in DCM, 6-G treatment enhances the Nrf2/HO-1 while reducing ferroptosis and inflammation. Since the Nrf2/HO-1 pathway in 6-G intervention DCM was not silenced in this study before ferroptosis and inflammation were observed, the exact relationship between Nrf2/HO-1 pathway and ferroptosis and inflammation needs further experimental verification, which is also the deficiency of this study.

## 5. Conclusion

In this study, we show that 6-G protects against DCM by reducing ferroptosis and inflammation via enhancing the Nrf2/HO-1 pathway. We suggest that 6-G may be a promising therapy or adjuvant drug for DCM.

## Figures and Tables

**Table 1:** Primers that were used in this study.

Target	Forward	Reverse
Mouse-FACL4	AGAGTCCAAAGCGAGGGAGA	TCTCTCCAGTTCCCAAAGGC
Mouse-GPX4	CCTCTGCTGCAAGAGCCTCCC	CTTATCCAGGCAGACCATGTGC
Mouse-IL-1*β*	TGGTACATCAGCCCGAAC	GTCAGCTGGATAGCGACA
Mouse-IL-6	GTCAGCTGGATAGCGACA	GAAGCACAGGAGCAGGTGTAGA
Mouse-TNF-*α*	GACATGCCGCCTGGAGAAAC	AGCCCAGGATGCCCTTTAGT
Mouse-Nrf2	GCCTTCCTCTGCTGCCATTAGTC	TGCCTTCAGTGTGCTTCTGGTTG
Mouse-HO-1	TGACACCTGAGGTCAAGCAC	GTCTCTGCAGGGGCAGTATC
Mouse-GAPDH	CCTTCATTGACCTCAACTACATGG	CTCGCTCCTGGAAGATGGTG
Rat-FACL4	TGGGCTGACAGAATCATGCG	AACTGTATAACCACCTTCCTGC
Rat-GPX4	CCTGGCTGGCACCATGT	CACACGCAACCCCTGTACTT
Rat-IL-1*β*	CCGTGGACCTTCCAGGATGA	GGGAACGTCACACACCAGCA
Rat-IL-6	GTTGCCTTCTTGGGACTGATG	GAAGTCTCCTCTCCGGACTTGT
Rat-TNF-*α*	GATTTGGTGACCAGGCTGTC	ATGACCCGTAGGGCGATT
Rat-Nrf2	TGTAGATGACCATGAGTCGC	TCCTGCCAAACTTGCTCCAT
Rat-HO-1	TGCTCGCATGAACACTCTGGAGAT	ATGGCATAAATTCCCACTGCCACG
Rat-GAPDH	GCAAGTTCAACGGCACAG	GCCAGTAGACTCCACGACAT

**Table 2:** Effects of 6-gingerol on blood glucose, heart weight (HW), lung weight (LW), and body weight (BW).

	CON (*n* = 10)	6-G (*n* = 10)	STZ (*n* = 14)	STZ+6-G L (*n* = 14)	STZ+6-G H (*n* = 14)
Blood glucose(mM)	8.66 ± 1.26	9.45 ± 1.44	25.75 ± 4.69^#^	24.89 ± 3.62	22.56 ± 4.08^∗^
BW (g)	28.67 ± 0.41	27.69 ± 0.46	28.89 ± 0.74	27.23 ± 0.72	27.37 ± 0.50
HW (mg)	113.05 ± 2.11	112.45 ± 2.06	136.83 ± 4.45^#^	120.10 ± 2.35^∗^	118.88 ± 2.53^∗^
LW (mg)	150.81 ± 5.74	149.88 ± 5.10	173.47 ± 7.31^#^	160.05 ± 4.40	156.49 ± 2.36
HW/BW (mg/g)	3.92 ± 0.19	4.03 ± 0.25	4.74 ± 0.52^#^	4.41 ± 0.23^∗^	4.34 ± 0.30^∗^
LW/BW (mg/g)	5.22 ± 0.37	5.39 ± 0.30	6.12 ± 0.55^#^	5.72 ± 0.48^∗^	5.60 ± 0.40^∗^

The blood glucose mentioned above is random blood glucose. Data are presented as means ± SEM; ^#^*p* < 0.05, compared with the CON or 6-G group; ^∗^*p* < 0.05, compared with the STZ group.

**Table 3:** Effects of 6-gingerol on echocardiographic parameters.

	CON (*n* = 10)	6-G (*n* = 10)	STZ (*n* = 14)	STZ+6-G L (*n* = 14)	STZ+6-G H (*n* = 14)
HR (bpm)	423.6 ± 6.60	428.7 ± 8.11	421.8 ± 9.32	426.7 ± 7.78	418.9 ± 12.32
LVPWD (mm)	0.83 ± 0.04	0.86 ± 0.03	0.78 ± 0.29	0.81 ± 0.03	0.84 ± 0.04
LVPWS (mm)	1.29 ± 0.04	1.35 ± 0.11	0.94 ± 0.04^#^	1.04 ± 0.03	1.12 ± 0.03^∗^
LVIDD (mm)	3.68 ± 0.12	3.64 ± 0.12	4.06 ± 0.11^#^	3.70 ± 0.06^∗^	3.64 ± 0.10^∗^
LVIDS (mm)	2.58 ± 0.09	2.60 ± 0.16	3.23 ± 0.09^#^	2.75 ± 0.05^∗^	2.63 ± 0.11^∗^
EF (%)	60.25 ± 1.30	57.91 ± 3.31	39.16 ± 2.22^#^	49.22 ± 1.88^∗^	53.17 ± 2.87^∗^
FS (%)	31.56 ± 0.90	30.22 ± 2.40	18.78 ± 1.23^#^	24.41 ± 1.15^∗^	26.97 ± 1.80^∗^

HR: heart rate; LVEDd: left ventricle end-diastolic dimension; LVEDs: left ventricle end-systolic dimension; LVIDd: left ventricular diastolic diameter; LVIDs: left ventricular systolic diameter; LVPWd: end-diastolic left ventricle posterior wall thickness; LVPWs: end-systolic left ventricle posterior wall thickness; EF: left ventricle ejection fraction; FS: fractional shortening. All values are expressed as mean ± SEM. ^#^*p* < 0.05, compared with the CON or 6-G group; ^∗^*p* < 0.05, compared with the STZ group.

## Data Availability

The data used to support the findings of this study are included within the article.

## Abbreviations

AGEs: Advanced glycation end products
DM: Diabetes mellitus
DCM: Diabetic cardiomyopathy
Nrf2: Nuclear factor erythroid 2-related factor 2
ROS: Reactive oxygen species
STZ: Streptozotocin
6-G: 6-Gingerol.

## References

[1] Wild S., Roglic G., Green A., Sicree R., King H. (2004). Global prevalence of diabetes. *Diabetes Care*.

[2] Grundy S. M., Benjamin I. J., Burke G. L. (1999). Diabetes and cardiovascular disease. *Circulation*.

[3] Yap J., Tay W. T., Teng T. K. (2019). Association of diabetes mellitus on cardiac remodeling, quality of life, and clinical outcomes in heart failure with reduced and preserved ejection fraction. *Journal of the American Heart Association*.

[4] Tate M., Grieve D. J., Ritchie R. H. (2017). Are Targeted Therapies for Diabetic Cardiomyopathy on the Horizon? Clinical Science. *Clinical Science*.

[5] Brunvand L., Heier M., Brunborg C. (2017). Advanced glycation end products in children with type 1 diabetes and early reduced diastolic heart function. *BMC Cardiovascular Disorders*.

[6] Avagimyan A., Popov S., Shalnova S. (2022). The pathophysiological basis of diabetic cardiomyopathy development. *Current Problems in Cardiology*.

[7] Wilson A. J., Gill E. K., Abudalo R. A., Edgar K. S., Watson C. J., Grieve D. J. (2018). Reactive oxygen species signalling in the diabetic heart: emerging prospect for therapeutic targeting. *Heart (British Cardiac Society)*.

[8] Liao H. H., Zhu J. X., Feng H. (2017). Myricetin possesses potential protective effects on diabetic cardiomyopathy through inhibiting I*κ*B*α*/NF*κ*B and enhancing Nrf2/HO-1. *Oxidative Medicine and Cellular Longevity*.

[9] Pradhan A. D., Manson J. E., Rifai N., Buring J. E., Ridker P. M. (2001). C-reactive protein, interleukin 6, and risk of developing type 2 diabetes mellitus. *Journal of the American Medical Association*.

[10] Dixon S. J., Lemberg K. M., Lamprecht M. R. (2012). Ferroptosis: an iron-dependent form of nonapoptotic cell death. *Cell*.

[11] Xiao Z., Kong B., Fang J. (2021). Ferrostatin-1 alleviates lipopolysaccharide-induced cardiac dysfunction. *Bioengineered*.

[12] Lin J. H., Yang K. T., Ting P. C. (2021). Gossypol acetic acid attenuates cardiac ischemia/reperfusion injury in rats via an antiferroptotic mechanism. *Biomolecules*.

[13] Liu Q., Sun L., Tan Y., Wang G., Lin X., Cai L. (2009). Role of iron deficiency and overload in the pathogenesis of diabetes and diabetic complications. *Current Medicinal Chemistry*.

[14] Swaminathan S., Fonseca V. A., Alam M. G., Shah S. V. (2007). The role of iron in diabetes and its complications. *Diabetes Care*.

[15] Palomer X., Salvadó L., Barroso E., Vázquez-Carrera M. (2013). An overview of the crosstalk between inflammatory processes and metabolic dysregulation during diabetic cardiomyopathy. *International Journal of Cardiology*.

[16] Cheng Z., Xiong X., Zhou Y. (2022). 6-Gingerol ameliorates metabolic disorders by inhibiting hypertrophy and hyperplasia of adipocytes in high-fat-diet induced obese mice. *Biomedicine & Pharmacotherapy = Biomedecine & Pharmacotherapie*.

[17] Rezazadeh-Shojaee F. S., Ramazani E., Kasaian J., Tayarani-Najaran Z. (2022). Protective effects of 6-gingerol on 6-hydroxydopamine-induced apoptosis in PC12 cells through modulation of SAPK/JNK and survivin activation. *Journal of Biochemical and Molecular Toxicology*.

[18] Manjunathan T., Guru A., Arokiaraj J., Gopinath P. (2021). 6-Gingerol and semisynthetic 6-gingerdione counteract oxidative stress induced by ROS in zebrafish. *Chemistry & Biodiversity*.

[19] Chen Z., Zhao Y., Song C. (2020). SanWeiGanJiang San relieves liver injury via Nrf2/Bach1. *Journal of Ethnopharmacology*.

[20] Hong M. K., Hu L. L., Zhang Y. X. (2020). 6-Gingerol ameliorates sepsis-induced liver injury through the Nrf2 pathway. *International Immunopharmacology*.

[21] Tsai Y., Xia C., Sun Z. (2020). The inhibitory effect of 6-gingerol on ubiquitin-specific peptidase 14 enhances autophagy-dependent ferroptosis and anti-tumor in vivo and in vitro. *Frontiers in Pharmacology*.

[22] El-Bassossy H. M., Elberry A. A., Ghareib S. A., Azhar A., Banjar Z. M., Watson M. L. (2016). Cardioprotection by 6-gingerol in diabetic rats. *Biochemical and Biophysical Research Communications*.

[23] Zhong X., Wang T., Xie Y. (2021). Activated protein C ameliorates diabetic cardiomyopathy via modulating OTUB1/YB-1/MEF2B axis. *Frontiers in Cardiovascular Medicine*.

[24] Wei J., Zhao Y., Liang H., Du W., Wang L. (2022). Preliminary evidence for the presence of multiple forms of cell death in diabetes cardiomyopathy. *Acta Pharmaceutica Sinica B*.

[25] Ju S. A., Nguyen Q. T., Nguyen T. H. T. (2021). Pretreatment with 6-gingerol ameliorates sepsis-induced immune dysfunction by regulating the cytokine balance and reducing lymphocyte apoptosis. *Oxidative Medicine and Cellular Longevity*.

[26] Wang X., Chen X., Zhou W. (2022). Ferroptosis is essential for diabetic cardiomyopathy and is prevented by sulforaphane via AMPK/NRF2 pathways. *Acta Pharmaceutica Sinica B*.

[27] Zou C., Liu X., Xie R. (2017). Deferiprone attenuates inflammation and myocardial fibrosis in diabetic cardiomyopathy rats. *Biochemical and Biophysical Research Communications*.

[28] Zheng Y., Li X. K., Wang Y., Cai L. (2008). The role of zinc, copper and iron in the pathogenesis of diabetes and diabetic complications: therapeutic effects by chelators. *Hemoglobin*.

[29] Tan Y., Zhang Z., Zheng C., Wintergerst K. A., Keller B. B., Cai L. (2020). Mechanisms of diabetic cardiomyopathy and potential therapeutic strategies: preclinical and clinical evidence. *Nature Reviews Cardiology*.

[30] Minotti G., Aust S. D. (1987). The role of iron in the initiation of lipid peroxidation. *Chemistry and Physics of Lipids*.

[31] Chen X., Li J., Kang R., Klionsky D. J., Tang D. (2021). Ferroptosis: machinery and regulation. *Autophagy*.

[32] Doll S., Proneth B., Tyurina Y. Y. (2017). ACSL4 dictates ferroptosis sensitivity by shaping cellular lipid composition. *Nature Chemical Biology*.

[33] Seibt T. M., Proneth B., Conrad M. (2019). Role of GPX4 in ferroptosis and its pharmacological implication. *Free Radical Biology & Medicine*.

[34] Sun X., Sun X., Meng H. (2022). Krill oil inhibits NLRP3 inflammasome activation in the prevention of the pathological injuries of diabetic cardiomyopathy. *Nutrients*.

[35] Zhang C., Han M., Zhang X., Tong H., Sun X., Sun G. (2022). Ginsenoside Rb1 protects against diabetic cardiomyopathy by regulating the adipocytokine pathway. *Journal of Inflammation Research*.

[36] Luo J., Chen J., Yang C. (2021). 6-Gingerol protects against cerebral ischemia/reperfusion injury by inhibiting NLRP3 inflammasome and apoptosis via TRPV1 / FAF1 complex dissociation- mediated autophagy. *International Immunopharmacology*.

[37] Almatroodi S. A., Alnuqaydan A. M., Babiker A. Y., Almogbel M. A., Khan A. A., Husain R. A. (2021). 6-Gingerol, a bioactive compound of ginger attenuates renal damage in streptozotocin-induced diabetic rats by regulating the oxidative stress and inflammation. *Pharmaceutics*.

[38] Xu L., Chen R., Zhang X. (2021). Scutellarin protects against diabetic cardiomyopathy via inhibiting oxidative stress and inflammatory response in mice. *Annals of Palliative Medicine*.

[39] Li X., Wu Y., Zhao J. (2020). Distinct cardiac energy metabolism and oxidative stress adaptations between obese and non-obese type 2 diabetes mellitus. *Theranostics*.

[40] Lian Y., Xia X., Zhao H., Zhu Y. (2017). The potential of chrysophanol in protecting against high fat-induced cardiac injury through Nrf2-regulated anti-inflammation, anti-oxidant and anti- fibrosis in Nrf2 knockout mice. *Biomedicine & Pharmacotherapy = Biomedecine & Pharmacotherapie*.

[41] Thimmulappa R. K., Lee H., Rangasamy T. (2006). Nrf2 is a critical regulator of the innate immune response and survival during experimental sepsis. *The Journal of Clinical Investigation*.

